# *mer*-Hydridotris(tri­methyl­phosphane-κ*P*)(d-valinato-κ^2^ *N*,*O*)iridium hexa­fluorido­phosphate di­chloro­methane 0.675-solvate

**DOI:** 10.1107/S1600536814002165

**Published:** 2014-02-05

**Authors:** Joseph S. Merola, Carla Slebodnick, Michael Berg, Melissa K. Ritchie

**Affiliations:** a Department of Chemistry, Virginia Tech, Blacksburg, VA 24061, USA

## Abstract

The title compound, [Ir(C_5_H_10_NO_2_)H(C_3_H_9_P)_3_]PF_6_·0.675CH_2_Cl_2_, an iridium compound with a meridional arrangement of PMe_3_ groups, *O*,*N*-bidentate coordination of d-valine and with a hydride ligand *trans* to the N atom is compared with the l-valine complex reported previously. As expected, the complexes from the corresponding l and d isomers of valine crystallize in enanti­omorphic space groups (*P*4_3_ and *P*4_1_, respectively). In the crystal, N—H⋯O and N—H⋯F hydrogen bonding is observed, the N—H to carbonyl oxygen hydrogen bond producing a helical motif that proceeds along the 4_1_ screw of the *c* axis.

## Related literature   

The structure of the related l-valine complex has been described by Roy *et al.* (2006 ▶). For studies of hydrogen-bonded lattice systems that lose crystallinity on loss of solvent and an analogous one that retains crystallinity, see: Parkin & Behrman (2009 ▶, 2011 ▶). An analysis of the geometric paramaters for hydrogen bonds is given by Wood *et al.* (2009 ▶). 
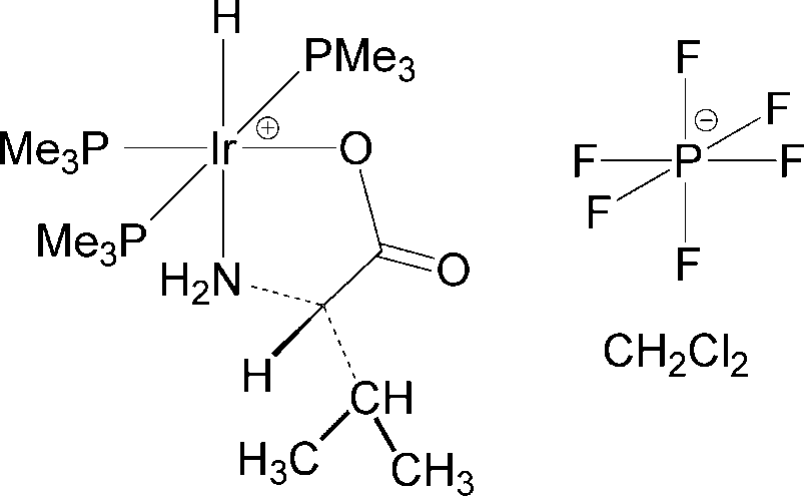


## Experimental   

### 

#### Crystal data  

[Ir(C_5_H_10_NO_2_)H(C_3_H_9_P)_3_]PF_6_·0.675CH_2_Cl_2_
*M* *_r_* = 739.86
Tetragonal, 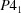

*a* = 14.04454 (17) Å
*c* = 14.2657 (3) Å
*V* = 2813.89 (9) Å^3^
*Z* = 4
Mo *K*α radiation
μ = 5.15 mm^−1^
*T* = 100 K
0.17 × 0.05 × 0.05 mm

#### Data collection  

Oxford Diffraction Xcalibur2 (Eos, Gemini ultra) diffractometer
Absorption correction: gaussian (*CrysAlis PRO*; Oxford Diffraction, 2010 ▶) *T*_min_ = 0.449, *T*_max_ = 0.774
39354 measured reflections
9371 independent reflections
7266 reflections with *I* > 2σ(*I*)
*R*_int_ = 0.069

#### Refinement  

*R*[*F* ^2^ > 2σ(*F* ^2^)] = 0.031
*wR*(*F* ^2^) = 0.046
*S* = 0.81
9371 reflections
301 parameters
2 restraints
H atoms treated by a mixture of independent and constrained refinement
Δρ_max_ = 0.70 e Å^−3^
Δρ_min_ = −0.61 e Å^−3^
Absolute structure: Flack parameter determined using 3004 quotients [(*I* ^+^)−(*I* ^−^)]/[(*I* ^+^)+(*I* ^−^)] (Parsons *et al.*, 2013 ▶)
Absolute structure parameter: −0.021 (4)

### 

Data collection: *CrysAlis PRO* (Oxford Diffraction, 2010 ▶); cell refinement: *CrysAlis PRO*; data reduction: *CrysAlis PRO*; program(s) used to solve structure: *SHELXS97* (Sheldrick, 2008 ▶); program(s) used to refine structure: *SHELXL97* (Sheldrick, 2008 ▶); molecular graphics: *OLEX2* (Dolomanov *et al.*, 2009 ▶); software used to prepare material for publication: *OLEX2*.

## Supplementary Material

Crystal structure: contains datablock(s) I. DOI: 10.1107/S1600536814002165/pk2516sup1.cif

Structure factors: contains datablock(s) I. DOI: 10.1107/S1600536814002165/pk2516Isup2.hkl

Supporting information file. DOI: 10.1107/S1600536814002165/pk2516Isup3.mol

CCDC reference:

Additional supporting information:  crystallographic information; 3D view; checkCIF report

## Figures and Tables

**Table 1:** Hydrogen-bond geometry (Å, °)

*D*—H⋯*A*	*D*—H	H⋯*A*	*D*⋯*A*	*D*—H⋯*A*
N1—H1*A*⋯F1	0.92 (6)	2.39 (6)	3.157 (7)	140 (5)
N1—H1*B*⋯O2^i^	0.83 (6)	2.02 (6)	2.848 (7)	172 (6)

Symmetry code: (i) 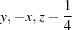.

**Fig. 1.**
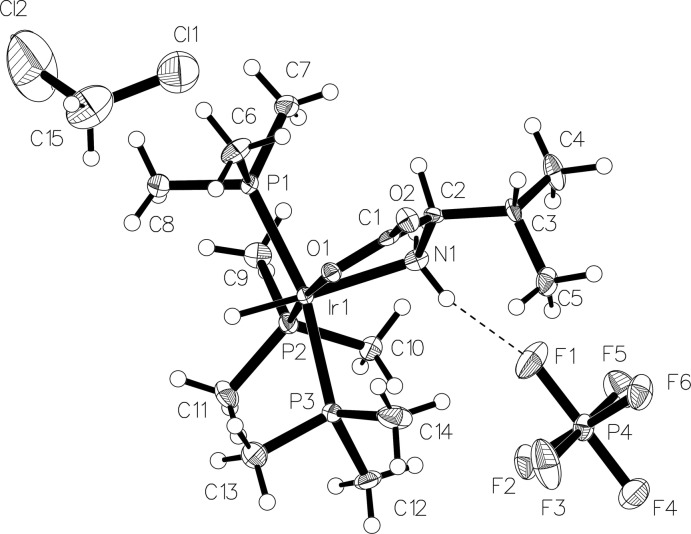
Thermal ellipsoid plot of the title compound showing the ellipsoids at the 50% probability level. The dichloromethane of solvation has a site occupancy of ~68%.

**Fig. 2.**
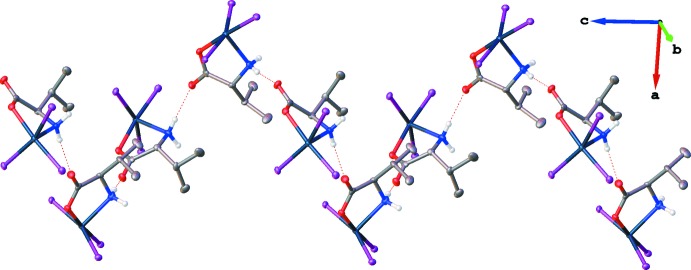
A view of the hydrogen-bonded extended helical lattice of the title compound. Ellipsoids are shown at the 50% probability level and hydrogen atoms as well as phosphorus methyl atoms are omitted for clarity.

**Fig. 3.**
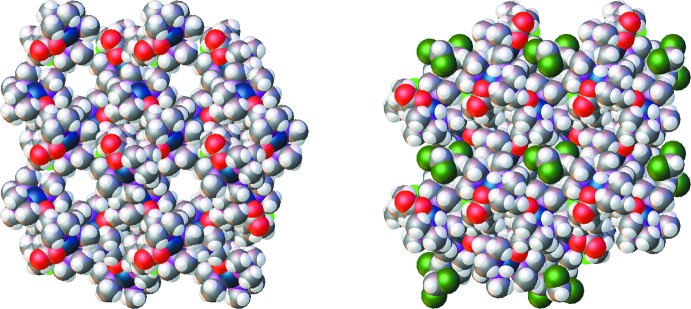
A space filling model of both the D– and L– valine iridium complexes packed from -0.5 → 1.0 along a, -0.5 → 1.0 along b, and -0.5→ 1.0 along c. The L–valine structure (left) shows a void while the D–valine structure (right) shows the dichloromethane in the lattice (68% occupancy). Spheres are shown at full van der Waals radii.

## supplementary crystallographic information

### 1. Comment

Both the *D*– and *L*-Valine complexes of the type [HIr(AA)(PMe_3_)_3_][PF_6_] crystallize in primitive tetragonal space groups, with the *L*–valine complex in P4_3_ and the D-valine complex in the enantiomorphic group P4_1_. The former structure was measured at room temperature on a Siemens P4 diffractometer while the latter was measured on an Oxford Diffraction instrument at 100 K. Accounting for room temperature *vs.* 100 K, the unit-cell parameters are essentially the same. Figure 1 shows a thermal ellipsoid plot of the asymmetric unit of the title compound. N—H to carbonyl oxygen hydrogen bonding produces a helical motif that proceeds along the 4_1_ screw of the *c* axis. The helical motif for the H-bonding is shown in figure 2. Hydrogen bonding also occurs between the second N—H atom and a fluorine atom of the PF_6_^-^ anion. Table 1 lists the hydrogen bonding parameters for the hydrogen bonds N1—H1A···F1 and N1—H1B···O2. With an H···O distance of 2.02 (6) Å and an N—H···O angle of 172 (6)°, the N1—H1B···O bond is a strong hydrogen bond while the N—H···F bond is not as strong based on geometric parameters, but still not a "weak" H-bond (Wood *et al.*, 2009).

In addition to the overall quality improvement of the structure of the *D* compound reported here at 100 K compared with the *L*–valine compound at RT, the issue of lattice solvent is an interesting one. In the previous report, the crystals were isolated and handled in air at room temperature for a period of days before mounting them and collecting data at room temperature. The *L*–valine complex showed very large voids (573 Å^3^) with negligible residual electron density. The current *D*–valine complex clearly shows dichloromethane within the structure, but each dichloromethane site is only ~68% occupied. Here, it would appear that dichloromethane of solvation is partially lost.

Often, for molecular compounds, loss of solvent of crystallization results in the collapse of the crystal lattice. The *D*–valine complex with partial loss and the *L*-valine with complete solvent loss maintain the crystal lattice structural integrity. Figure 3 shows a view of both the *D*– and *L*–valine space filling packing diagrams that show, the CH_2_Cl_2_ in the title compound and the empty space in the *L*–valine structure reported previously. The loss of solvent with preservation of the crystal lattice is the norm for metal-organic framework (MOF) compounds, but those involve strong coordination bonds between metals and linking ligands. Maintaining the crystal lattice solely with hydrogen bonding is not new, but it is somewhat rare. For another example, see Parkin and Behrman (2011).

### 2. Experimental

For a detailed description of the synthetic procedure for all of the tris-trimethylphosphine iridium amino acid complexes, see (Roy *et al.*, 2006). The title compound was recrystallized by the layering of diethyl ether over a dichloromethane solution. After several days of slow diffusion, suitable single crystals grew at the interface.

### 3. Refinement

The H-atoms on the amine nitrogen were located from the residual electron density map and the positions refined independently. The N—H bonds are 0.92 (6) and 0.83 (6) Å. The displacement parameters were fixed at *U*_iso_(H)=1.2Ueq(N1). The hydride was located in the residual electron density map, the distance restrained to 1.57 (2) Å, and the displacement parameter fixed at *U*_iso_(H1)=1.5U_eq_(Ir1). After locating the iridium hydride salt, additional strong residual electron density peaks were modeled as a partially occupied CH_2_Cl_2_ molecule with occupancy that refined to 0.675 (6).

### Crystal data

**Table:**

[Ir(C_5_H_10_NO_2_)H(C_3_H_9_P)_3_]PF_6_·0.675CH_2_Cl_2_	*D*_x_ = 1.746 Mg m^−^^3^
*M**_r_* = 739.86	Mo *K*α radiation, λ = 0.71073 Å
Tetragonal, *P*4_1_	Cell parameters from 12856 reflections
*a* = 14.04454 (17) Å	θ = 3.5–31.4°
*c* = 14.2657 (3) Å	µ = 5.15 mm^−^^1^
*V* = 2813.89 (9) Å^3^	*T* = 100 K
*Z* = 4	Needle, colourless
*F*(000) = 1457	0.17 × 0.05 × 0.05 mm

### Data collection

**Table:**

Oxford Diffraction Xcalibur2 (Eos, Gemini ultra) diffractometer	9371 independent reflections
Radiation source: Enhance (Mo) X-ray Source	7266 reflections with *I* > 2σ(*I*)
Graphite monochromator	*R*_int_ = 0.069
Detector resolution: 16.0122 pixels mm^-1^	θ_max_ = 31.5°, θ_min_ = 3.5°
ω scans	*h* = −19→20
Absorption correction: gaussian (*CrysAlis PRO*; Oxford Diffraction, 2010)	*k* = −20→20
*T*_min_ = 0.449, *T*_max_ = 0.774	*l* = −20→20
39354 measured reflections	

### Refinement

**Table:**

Refinement on *F*^2^	Hydrogen site location: mixed
Least-squares matrix: full	H atoms treated by a mixture of independent and constrained refinement
*R*[*F*^2^ > 2σ(*F*^2^)] = 0.031	*w* = 1/[σ^2^(*F*_o_^2^) + (0.0129*P*)^2^] where *P* = (*F*_o_^2^ + 2*F*_c_^2^)/3
*wR*(*F*^2^) = 0.046	(Δ/σ)_max_ = 0.001
*S* = 0.81	Δρ_max_ = 0.70 e Å^−^^3^
9371 reflections	Δρ_min_ = −0.61 e Å^−^^3^
301 parameters	Absolute structure: Flack parameter determined using 3004 quotients [(*I*^+^)-(*I*^-^)]/[(*I*^+^)+(*I*^-^)] (Parsons *et al.*, 2013)
2 restraints	Absolute structure parameter: −0.021 (4)

### Special details

**Table:**

Geometry. All e.s.d.'s (except the e.s.d. in the dihedral angle between two l.s. planes) are estimated using the full covariance matrix. The cell e.s.d.'s are taken into account individually in the estimation of e.s.d.'s in distances, angles and torsion angles; correlations between e.s.d.'s in cell parameters are only used when they are defined by crystal symmetry. An approximate (isotropic) treatment of cell e.s.d.'s is used for estimating e.s.d.'s involving l.s. planes.

### Fractional atomic coordinates and isotropic or equivalent isotropic displacement parameters (Å^2^)

**Table:**

	*x*	*y*	*z*	*U*_iso_*/*U*_eq_	Occ. (<1)
Ir1	0.35067 (2)	0.06307 (2)	0.87490 (2)	0.01215 (4)	
H	0.423 (3)	0.057 (4)	0.946 (3)	0.018*	
O1	0.2567 (3)	0.1552 (3)	0.9483 (3)	0.0147 (9)	
O2	0.1109 (3)	0.2137 (3)	0.9557 (3)	0.0185 (9)	
N1	0.2309 (4)	0.0780 (4)	0.7763 (4)	0.0167 (11)	
H1A	0.243 (4)	0.127 (4)	0.735 (4)	0.020*	
H1B	0.221 (4)	0.023 (5)	0.758 (4)	0.020*	
C1	0.1714 (4)	0.1635 (4)	0.9161 (4)	0.0139 (12)	
C2	0.1437 (4)	0.1072 (4)	0.8295 (4)	0.0131 (12)	
H2	0.1141	0.0469	0.8529	0.016*	
C3	0.0677 (4)	0.1561 (4)	0.7699 (4)	0.0161 (12)	
H3	0.0145	0.1728	0.8134	0.019*	
C4	0.0260 (5)	0.0889 (5)	0.6968 (4)	0.0288 (16)	
H4A	0.0754	0.0717	0.6512	0.043*	
H4B	−0.0268	0.1205	0.6643	0.043*	
H4C	0.0026	0.0312	0.7277	0.043*	
C5	0.1009 (4)	0.2493 (4)	0.7263 (4)	0.0249 (15)	
H5A	0.1337	0.2876	0.7737	0.037*	
H5B	0.0456	0.2845	0.7026	0.037*	
H5C	0.1446	0.2358	0.6744	0.037*	
P1	0.28924 (11)	−0.05993 (12)	0.96794 (11)	0.0148 (3)	
C6	0.2254 (5)	−0.0111 (5)	1.0676 (4)	0.0281 (17)	
H6A	0.2641	0.0385	1.0973	0.042*	
H6B	0.2126	−0.0619	1.1130	0.042*	
H6C	0.1651	0.0165	1.0463	0.042*	
C7	0.2042 (5)	−0.1472 (4)	0.9273 (4)	0.0255 (15)	
H7A	0.1438	−0.1156	0.9126	0.038*	
H7B	0.1938	−0.1949	0.9764	0.038*	
H7C	0.2289	−0.1785	0.8709	0.038*	
C8	0.3778 (4)	−0.1308 (5)	1.0273 (4)	0.0287 (16)	
H8A	0.4116	−0.1703	0.9815	0.043*	
H8B	0.3467	−0.1718	1.0737	0.043*	
H8C	0.4233	−0.0888	1.0590	0.043*	
P2	0.44500 (11)	−0.02850 (12)	0.78431 (11)	0.0161 (3)	
C9	0.4335 (5)	−0.1576 (4)	0.7880 (4)	0.0230 (13)	
H9A	0.4649	−0.1853	0.7330	0.034*	
H9B	0.3660	−0.1749	0.7877	0.034*	
H9C	0.4636	−0.1819	0.8451	0.034*	
C10	0.4244 (4)	−0.0090 (4)	0.6599 (4)	0.0190 (14)	
H10A	0.4285	0.0592	0.6461	0.028*	
H10B	0.3609	−0.0325	0.6430	0.028*	
H10C	0.4726	−0.0433	0.6234	0.028*	
C11	0.5723 (4)	−0.0150 (5)	0.8001 (4)	0.0252 (15)	
H11A	0.6059	−0.0491	0.7502	0.038*	
H11B	0.5908	−0.0412	0.8611	0.038*	
H11C	0.5889	0.0527	0.7976	0.038*	
P3	0.42554 (11)	0.20679 (11)	0.83341 (11)	0.0156 (3)	
C12	0.4899 (4)	0.2255 (5)	0.7248 (4)	0.0246 (15)	
H12A	0.5181	0.2893	0.7249	0.037*	
H12B	0.4460	0.2196	0.6717	0.037*	
H12C	0.5405	0.1778	0.7190	0.037*	
C13	0.5114 (5)	0.2398 (5)	0.9217 (4)	0.0294 (17)	
H13A	0.5631	0.1929	0.9228	0.044*	
H13B	0.4803	0.2416	0.9832	0.044*	
H13C	0.5375	0.3028	0.9071	0.044*	
C14	0.3453 (5)	0.3082 (5)	0.8334 (6)	0.037 (2)	
H14A	0.3821	0.3670	0.8259	0.056*	
H14B	0.3104	0.3102	0.8928	0.056*	
H14C	0.3001	0.3020	0.7814	0.056*	
P4	0.29616 (12)	0.22465 (12)	0.48790 (11)	0.0201 (4)	
F1	0.2682 (3)	0.1526 (3)	0.5708 (3)	0.0469 (12)	
F2	0.4001 (3)	0.1782 (3)	0.4853 (3)	0.0322 (9)	
F3	0.3308 (3)	0.2988 (3)	0.5639 (3)	0.0554 (14)	
F4	0.3246 (3)	0.2950 (3)	0.4063 (3)	0.0439 (12)	
F5	0.2601 (3)	0.1496 (3)	0.4133 (3)	0.0426 (11)	
F6	0.1918 (3)	0.2709 (3)	0.4920 (3)	0.0353 (10)	
C15	0.5610 (11)	0.3894 (9)	0.4795 (10)	0.066 (4)	0.675 (6)
H15A	0.5070	0.3578	0.4475	0.080*	0.675 (6)
H15B	0.5730	0.3546	0.5387	0.080*	0.675 (6)
Cl1	0.6642 (3)	0.3794 (2)	0.4068 (3)	0.0708 (15)	0.675 (6)
Cl2	0.5294 (4)	0.4995 (4)	0.5055 (7)	0.157 (3)	0.675 (6)

### Atomic displacement parameters (Å^2^)

**Table:**

	*U*^11^	*U*^22^	*U*^33^	*U*^12^	*U*^13^	*U*^23^
Ir1	0.01075 (12)	0.01395 (12)	0.01177 (7)	−0.00014 (11)	−0.00084 (10)	0.00094 (10)
O1	0.015 (2)	0.015 (2)	0.014 (2)	−0.0025 (18)	−0.0022 (17)	−0.0019 (16)
O2	0.016 (2)	0.023 (2)	0.017 (2)	0.0037 (19)	0.0020 (17)	−0.0052 (18)
N1	0.020 (3)	0.013 (3)	0.017 (3)	0.001 (2)	0.004 (2)	−0.002 (2)
C1	0.016 (3)	0.012 (3)	0.014 (3)	−0.001 (2)	0.005 (2)	0.004 (2)
C2	0.010 (3)	0.012 (3)	0.017 (3)	−0.003 (2)	0.000 (2)	0.000 (2)
C3	0.010 (3)	0.024 (3)	0.015 (3)	0.003 (2)	−0.003 (2)	−0.001 (3)
C4	0.022 (4)	0.032 (4)	0.033 (4)	0.015 (3)	−0.010 (3)	−0.010 (3)
C5	0.020 (4)	0.029 (4)	0.025 (3)	0.011 (3)	0.002 (3)	0.006 (3)
P1	0.0151 (9)	0.0175 (9)	0.0118 (7)	−0.0009 (7)	−0.0008 (6)	0.0031 (6)
C6	0.037 (4)	0.024 (4)	0.023 (4)	0.001 (3)	0.011 (3)	0.006 (3)
C7	0.029 (4)	0.025 (4)	0.023 (3)	−0.010 (3)	−0.005 (3)	0.004 (3)
C8	0.020 (4)	0.038 (4)	0.029 (4)	−0.004 (3)	−0.003 (3)	0.013 (3)
P2	0.0127 (8)	0.0188 (9)	0.0166 (8)	0.0015 (7)	−0.0005 (6)	0.0011 (7)
C9	0.026 (4)	0.019 (4)	0.024 (3)	0.000 (3)	0.002 (3)	−0.006 (3)
C10	0.018 (3)	0.021 (3)	0.017 (3)	0.006 (3)	0.005 (2)	0.001 (2)
C11	0.014 (3)	0.034 (4)	0.027 (4)	0.004 (3)	−0.002 (3)	−0.002 (3)
P3	0.0137 (8)	0.0155 (8)	0.0177 (8)	−0.0013 (7)	−0.0004 (6)	0.0013 (6)
C12	0.028 (4)	0.026 (4)	0.019 (3)	−0.011 (3)	0.003 (3)	0.006 (3)
C13	0.036 (4)	0.027 (4)	0.025 (4)	−0.010 (3)	−0.010 (3)	−0.001 (3)
C14	0.023 (4)	0.018 (4)	0.072 (6)	0.000 (3)	0.005 (4)	0.004 (4)
P4	0.0213 (9)	0.0208 (9)	0.0180 (8)	0.0069 (7)	−0.0001 (7)	0.0002 (7)
F1	0.049 (3)	0.049 (3)	0.043 (3)	0.026 (2)	0.021 (2)	0.025 (2)
F2	0.026 (2)	0.039 (3)	0.031 (2)	0.0158 (19)	−0.0018 (17)	0.0012 (19)
F3	0.061 (3)	0.048 (3)	0.058 (3)	0.019 (2)	−0.034 (3)	−0.028 (2)
F4	0.040 (3)	0.046 (3)	0.046 (3)	0.002 (2)	0.004 (2)	0.021 (2)
F5	0.039 (3)	0.039 (3)	0.049 (3)	0.003 (2)	−0.008 (2)	−0.015 (2)
F6	0.032 (2)	0.042 (3)	0.031 (2)	0.017 (2)	0.0028 (18)	0.0063 (19)
C15	0.089 (12)	0.036 (8)	0.074 (11)	0.011 (7)	−0.013 (9)	0.001 (7)
Cl1	0.084 (3)	0.047 (2)	0.082 (3)	−0.0090 (19)	0.017 (2)	−0.0030 (18)
Cl2	0.140 (5)	0.061 (4)	0.270 (8)	0.039 (4)	0.095 (7)	0.018 (4)

### Geometric parameters (Å, º)

**Table:**

Ir1—H	1.44 (3)	P2—C9	1.821 (6)
Ir1—O1	2.124 (4)	P2—C10	1.820 (5)
Ir1—N1	2.203 (6)	P2—C11	1.811 (6)
Ir1—P1	2.3430 (16)	C9—H9A	0.9800
Ir1—P2	2.2537 (16)	C9—H9B	0.9800
Ir1—P3	2.3517 (16)	C9—H9C	0.9800
O1—C1	1.288 (7)	C10—H10A	0.9800
O2—C1	1.240 (6)	C10—H10B	0.9800
N1—H1A	0.92 (6)	C10—H10C	0.9800
N1—H1B	0.83 (6)	C11—H11A	0.9800
N1—C2	1.499 (7)	C11—H11B	0.9800
C1—C2	1.518 (7)	C11—H11C	0.9800
C2—H2	1.0000	P3—C12	1.813 (6)
C2—C3	1.528 (7)	P3—C13	1.805 (6)
C3—H3	1.0000	P3—C14	1.816 (7)
C3—C4	1.524 (8)	C12—H12A	0.9800
C3—C5	1.522 (8)	C12—H12B	0.9800
C4—H4A	0.9800	C12—H12C	0.9800
C4—H4B	0.9800	C13—H13A	0.9800
C4—H4C	0.9800	C13—H13B	0.9800
C5—H5A	0.9800	C13—H13C	0.9800
C5—H5B	0.9800	C14—H14A	0.9800
C5—H5C	0.9800	C14—H14B	0.9800
P1—C6	1.815 (6)	C14—H14C	0.9800
P1—C7	1.807 (6)	P4—F1	1.605 (4)
P1—C8	1.805 (6)	P4—F2	1.599 (4)
C6—H6A	0.9800	P4—F3	1.580 (4)
C6—H6B	0.9800	P4—F4	1.578 (4)
C6—H6C	0.9800	P4—F5	1.582 (4)
C7—H7A	0.9800	P4—F6	1.604 (4)
C7—H7B	0.9800	C15—H15A	0.9900
C7—H7C	0.9800	C15—H15B	0.9900
C8—H8A	0.9800	C15—Cl1	1.789 (15)
C8—H8B	0.9800	C15—Cl2	1.651 (13)
C8—H8C	0.9800		
			
O1—Ir1—H	97 (2)	H8A—C8—H8C	109.5
O1—Ir1—N1	77.43 (17)	H8B—C8—H8C	109.5
O1—Ir1—P1	86.61 (11)	C9—P2—Ir1	120.0 (2)
O1—Ir1—P2	174.55 (11)	C10—P2—Ir1	112.3 (2)
O1—Ir1—P3	83.06 (11)	C10—P2—C9	99.4 (3)
N1—Ir1—H	174 (2)	C11—P2—Ir1	116.7 (2)
N1—Ir1—P1	98.66 (15)	C11—P2—C9	100.9 (3)
N1—Ir1—P2	97.88 (14)	C11—P2—C10	105.2 (3)
N1—Ir1—P3	95.69 (15)	P2—C9—H9A	109.5
P1—Ir1—H	80 (2)	P2—C9—H9B	109.5
P1—Ir1—P3	160.05 (5)	P2—C9—H9C	109.5
P2—Ir1—H	88 (2)	H9A—C9—H9B	109.5
P2—Ir1—P1	96.93 (5)	H9A—C9—H9C	109.5
P2—Ir1—P3	94.74 (6)	H9B—C9—H9C	109.5
P3—Ir1—H	85 (2)	P2—C10—H10A	109.5
C1—O1—Ir1	117.2 (3)	P2—C10—H10B	109.5
Ir1—N1—H1A	110 (4)	P2—C10—H10C	109.5
Ir1—N1—H1B	104 (4)	H10A—C10—H10B	109.5
H1A—N1—H1B	122 (6)	H10A—C10—H10C	109.5
C2—N1—Ir1	109.1 (4)	H10B—C10—H10C	109.5
C2—N1—H1A	106 (4)	P2—C11—H11A	109.5
C2—N1—H1B	106 (4)	P2—C11—H11B	109.5
O1—C1—C2	118.8 (5)	P2—C11—H11C	109.5
O2—C1—O1	121.8 (5)	H11A—C11—H11B	109.5
O2—C1—C2	119.4 (5)	H11A—C11—H11C	109.5
N1—C2—C1	110.2 (5)	H11B—C11—H11C	109.5
N1—C2—H2	106.1	C12—P3—Ir1	124.2 (2)
N1—C2—C3	114.3 (5)	C12—P3—C14	101.3 (3)
C1—C2—H2	106.1	C13—P3—Ir1	110.1 (2)
C1—C2—C3	113.5 (5)	C13—P3—C12	103.1 (3)
C3—C2—H2	106.1	C13—P3—C14	102.3 (3)
C2—C3—H3	106.3	C14—P3—Ir1	113.3 (2)
C4—C3—C2	111.8 (5)	P3—C12—H12A	109.5
C4—C3—H3	106.3	P3—C12—H12B	109.5
C5—C3—C2	113.7 (5)	P3—C12—H12C	109.5
C5—C3—H3	106.3	H12A—C12—H12B	109.5
C5—C3—C4	111.8 (5)	H12A—C12—H12C	109.5
C3—C4—H4A	109.5	H12B—C12—H12C	109.5
C3—C4—H4B	109.5	P3—C13—H13A	109.5
C3—C4—H4C	109.5	P3—C13—H13B	109.5
H4A—C4—H4B	109.5	P3—C13—H13C	109.5
H4A—C4—H4C	109.5	H13A—C13—H13B	109.5
H4B—C4—H4C	109.5	H13A—C13—H13C	109.5
C3—C5—H5A	109.5	H13B—C13—H13C	109.5
C3—C5—H5B	109.5	P3—C14—H14A	109.5
C3—C5—H5C	109.5	P3—C14—H14B	109.5
H5A—C5—H5B	109.5	P3—C14—H14C	109.5
H5A—C5—H5C	109.5	H14A—C14—H14B	109.5
H5B—C5—H5C	109.5	H14A—C14—H14C	109.5
C6—P1—Ir1	110.3 (2)	H14B—C14—H14C	109.5
C7—P1—Ir1	124.2 (2)	F2—P4—F1	89.1 (2)
C7—P1—C6	100.4 (3)	F2—P4—F6	179.2 (2)
C8—P1—Ir1	114.8 (2)	F3—P4—F1	89.1 (3)
C8—P1—C6	100.5 (3)	F3—P4—F2	90.2 (2)
C8—P1—C7	103.4 (3)	F3—P4—F5	178.9 (3)
P1—C6—H6A	109.5	F3—P4—F6	89.4 (2)
P1—C6—H6B	109.5	F4—P4—F1	179.5 (2)
P1—C6—H6C	109.5	F4—P4—F2	90.4 (2)
H6A—C6—H6B	109.5	F4—P4—F3	90.9 (3)
H6A—C6—H6C	109.5	F4—P4—F5	90.1 (2)
H6B—C6—H6C	109.5	F4—P4—F6	90.3 (2)
P1—C7—H7A	109.5	F5—P4—F1	89.8 (2)
P1—C7—H7B	109.5	F5—P4—F2	90.3 (2)
P1—C7—H7C	109.5	F5—P4—F6	90.1 (2)
H7A—C7—H7B	109.5	F6—P4—F1	90.3 (2)
H7A—C7—H7C	109.5	H15A—C15—H15B	107.5
H7B—C7—H7C	109.5	Cl1—C15—H15A	108.5
P1—C8—H8A	109.5	Cl1—C15—H15B	108.5
P1—C8—H8B	109.5	Cl2—C15—H15A	108.5
P1—C8—H8C	109.5	Cl2—C15—H15B	108.5
H8A—C8—H8B	109.5	Cl2—C15—Cl1	115.0 (8)

### Hydrogen-bond geometry (Å, º)

**Table:**

*D*—H···*A*	*D*—H	H···*A*	*D*···*A*	*D*—H···*A*
N1—H1*A*···F1	0.92 (6)	2.39 (6)	3.157 (7)	140 (5)
N1—H1*B*···O2^i^	0.83 (6)	2.02 (6)	2.848 (7)	172 (6)

Symmetry code: (i) *y*, −*x*, *z*−1/4.
